# Poly[[tetra­kis­(μ-1,2-di-4-pyridyl­ethyl­ene-κ^2^ *N*:*N*′)tetra­kis­(seleno­cyanato-κ*N*)dimanganese(II)] 1,2-di-4-pyridyl­ethyl­ene disolvate]

**DOI:** 10.1107/S1600536811016199

**Published:** 2011-05-07

**Authors:** Susanne Wöhlert, Inke Jess, Christian Näther

**Affiliations:** a Institut für Anorganische Chemie, Christian-Albrechts-Universität Kiel, Max-Eyth-Strasse 2, 24118 Kiel, Germany

## Abstract

The crystal structure of the title compound, {[Mn(NCSe)_2_(C_12_H_10_N_2_)_2_]·C_12_H_10_N_2_}_2*n*_, consists of two crystallographically independent Mn cations, four seleno­cyanate anions, four 1,2-di-pyridylethylene (bpe) ligands (two of which are located on centers of inversion) and two bpe solvent molecules. Each manganese(II) cation is coordinated by two terminally *N*-bonded seleno­cyanate anions and four bpe ligands within a slightly distorted octa­hedron. The manganese(II) cations are linked by the bpe ligands into chains that are further connected by these ligands into layers. These layers are stacked, forming cavities in which additional bpe solvent molecules are embedded.

## Related literature

For background to this work, see: Boeckmann & Näther (2010 ▶); Wriedt *et al.* (2009 ▶); Wriedt & Näther (2010 ▶). For a description of the Cambridge Crystallographic Database, see: Allen (2002 ▶).
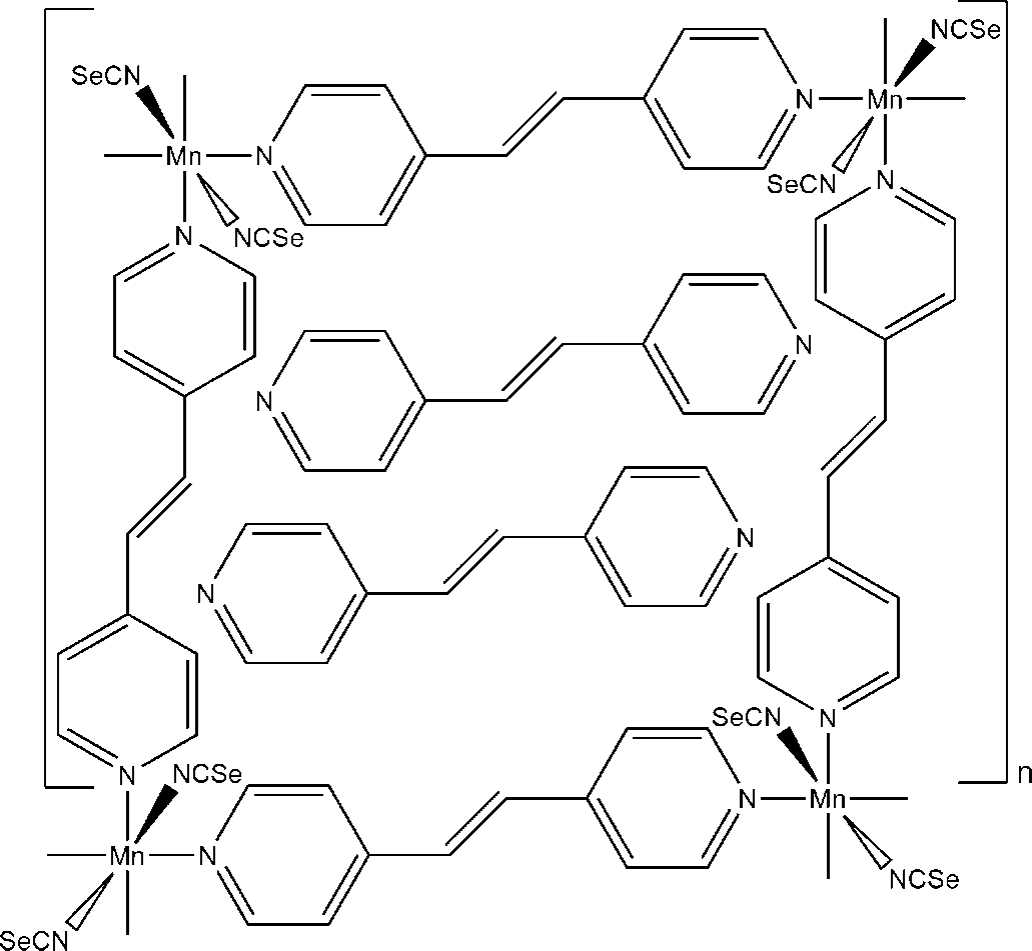


## Experimental

### 

#### Crystal data

[Mn_2_(NCSe)_4_(C_12_H_10_N_2_)_4_]·2C_12_H_10_N_2_
*M* *_r_* = 1623.12
Monoclinic, 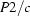

*a* = 14.3102 (3) Å
*b* = 13.9933 (2) Å
*c* = 36.0614 (6) Å
β = 91.211 (2)°
*V* = 7219.6 (2) Å^3^
*Z* = 4
Mo *K*α radiation
μ = 2.42 mm^−1^
*T* = 293 K
0.19 × 0.13 × 0.08 mm

#### Data collection

Stoe IPDS-2 diffractometer
Absorption correction: numerical (*X-SHAPE* and *X-RED32*; Stoe & Cie, 2008) ▶ *T*_min_ = 0.689, *T*_max_ = 0.853
68086 measured reflections
12826 independent reflections
10467 reflections with *I* > 2σ(*I*)
*R*_int_ = 0.054

#### Refinement

*R*[*F* ^2^ > 2σ(*F* ^2^)] = 0.056
*wR*(*F* ^2^) = 0.124
*S* = 1.10
12826 reflections
883 parameters
H-atom parameters constrained
Δρ_max_ = 0.79 e Å^−3^
Δρ_min_ = −0.78 e Å^−3^

### 

Data collection: *X-AREA* (Stoe & Cie, 2008) ▶; cell refinement: *X-AREA* ▶; data reduction: *X-AREA* ▶; program(s) used to solve structure: *SHELXS97* (Sheldrick, 2008 ▶); program(s) used to refine structure: *SHELXL97* (Sheldrick, 2008 ▶); molecular graphics: *XP* in *SHELXTL* (Sheldrick, 2008 ▶); software used to prepare material for publication: *XP* in *SHELXTL*and *DIAMOND* (Brandenburg, 2011 ▶).

## Supplementary Material

Crystal structure: contains datablocks I, global. DOI: 10.1107/S1600536811016199/bt5535sup1.cif

Structure factors: contains datablocks I. DOI: 10.1107/S1600536811016199/bt5535Isup2.hkl

Additional supplementary materials:  crystallographic information; 3D view; checkCIF report

## Figures and Tables

**Table 1:** Selected bond lengths (Å)

Mn2—N16	2.172 (4)
Mn2—N17	2.185 (3)
Mn2—N41	2.317 (3)
Mn2—N121	2.322 (3)
Mn2—N22	2.325 (3)
Mn1—N14	2.176 (3)
Mn1—N13	2.183 (4)
Mn1—N21	2.324 (3)
Mn1—N1	2.338 (3)
Mn1—N81	2.339 (3)

**Fig. 1.**
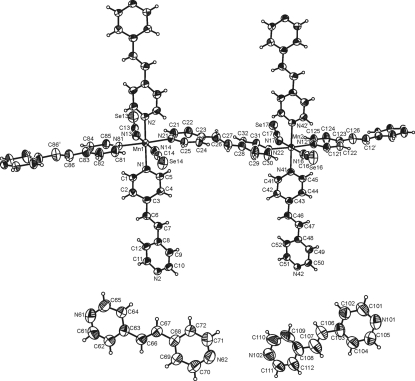
Crystal structure of the title compound showing the Mn coordination (top) and the noncoordinating bpe ligands (bottom) with labelling and displacement ellipsoids drawn at the 50% probability level. [Symmetry codes: (I) x, y+1, z; (ii) -x+2, y, -z+1/2; (iii) -x+1, y, -z-1/2.]

**Fig. 2.**
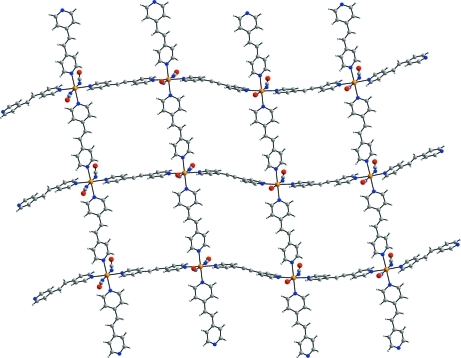
Crystal structure of the title compound with view onto the layers. The noncoordinated bpe ligands have been omitted for clearity.

**Fig. 3.**
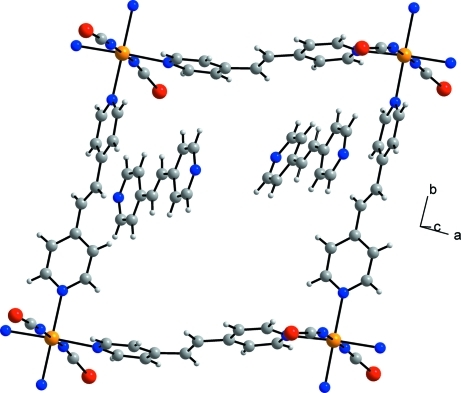
Crystal structure of the title compound with view approximately along the crystallographic c axis.

## supplementary crystallographic information

### Comment

In our recent work on the synthesis, structures and properties of new coordination polymers based on paramagnetic transition metals, thiocyanato or selenocyanato anions and *N*-donor ligands we have shown that ligand-deficient compounds can be prepared by thermal decomposition reactions (Wriedt *et al.*, 2009, Boeckmann & Näther, 2010 and Wriedt & Näther, 2010). Within this project we tried to prepare new ligand-rich precursor compounds based on manganese(II) selenocyanato and the *N*-donor ligand 1,2-bis(4-pyridyl)-ethylene (bpe) which resulted in the formation of the title compound that were identified by single crystal X-ray diffraction.

In the crystal structure of the title compound each managnese(II) cation is coordinated by two terminally *N*-bonded selenocyanato anions and four bridging bpe ligands (Fig. 1). The MnN_6_ octahedra are slightly distorted with distances in the range of 2.172 (4) Å to 2.339 (3) Å. The angles around the metal cations are in the range of 86.94 (12) ° to 179.89 (16) °. The manganese(II) cations are linked by the bpe ligands into chains that are further connected into layers by additional coligands (Fig. 2). The layers are stacked in order that cavities are formed, in which non-coordinated bpe molecules are located (Fig. 3). The Mn—Mn distances within the chains are in range of 13.9666 (8) Å to 13.9933 (8) Å.

It must be noted that according to a search in the CCDC database (ConQuest Ver.1.12.2010; Allen, 2002) compounds based on manganese(II) selenocyanate and 1,2-bis(4-pyridyl)-ethylene are unknown.

### Experimental

Manganese chloride tetrahydrate (MnCl_2_ ×  4H_2_O) and 1,2-bis(4-pyridyl)-ethylene (bpe) were obtained from sigma aldrich. Potassium selenocyanate (KNCSe) and acetonitrile (MeCN) were obtained from alfa aesar. All chemicals were used without further purification. 0.15 mmol (25.4 mg) MnCl_2_ x 4H_2_O, 0.3 mmol (43 mg) KNCSe and 0.6 mmol (107.5 mg) bpe were reacted with 1 ml MeCN in a closed test-tube at 120 ° C for three days. On cooling orange block-shaped single crystals of the title compound were obtained.

### Refinement

H atoms were positioned with idealized geometry and were refined isotropically with *U*_iso_(H) = 1.2*U*_eq_(C) and C—H distances of 0.93 Å using a riding model.

### Crystal data

**Table:**

[Mn_2_(NCSe)_4_(C_12_H_10_N_2_)_4_]·2C_12_H_10_N_2_	*F*(000) = 3256
*M**_r_* = 1623.12	*D*_x_ = 1.493 Mg m^−^^3^
Monoclinic, *P*2/*c*	Mo *K*α radiation, λ = 0.71073 Å
Hall symbol: -P 2yc	Cell parameters from 68086 reflections
*a* = 14.3102 (3) Å	θ = 1.1–25.2°
*b* = 13.9933 (2) Å	µ = 2.42 mm^−^^1^
*c* = 36.0614 (6) Å	*T* = 293 K
β = 91.211 (2)°	Block, orange
*V* = 7219.6 (2) Å^3^	0.19 × 0.13 × 0.08 mm
*Z* = 4	

### Data collection

**Table:**

Stoe IPDS-2 diffractometer	12826 independent reflections
Radiation source: fine-focus sealed tube	10467 reflections with *I* > 2σ(*I*)
graphite	*R*_int_ = 0.054
ω scans	θ_max_ = 25.2°, θ_min_ = 1.1°
Absorption correction: numerical (*X-SHAPE* and *X-RED32*; Stoe & Cie, 2008)	*h* = −17→17
*T*_min_ = 0.689, *T*_max_ = 0.853	*k* = −16→16
68086 measured reflections	*l* = −43→43

### Refinement

**Table:**

Refinement on *F*^2^	Primary atom site location: structure-invariant direct methods
Least-squares matrix: full	Secondary atom site location: difference Fourier map
*R*[*F*^2^ > 2σ(*F*^2^)] = 0.056	Hydrogen site location: inferred from neighbouring sites
*wR*(*F*^2^) = 0.124	H-atom parameters constrained
*S* = 1.10	*w* = 1/[σ^2^(*F*_o_^2^) + (0.0431*P*)^2^ + 9.7881*P*] where *P* = (*F*_o_^2^ + 2*F*_c_^2^)/3
12826 reflections	(Δ/σ)_max_ = 0.001
883 parameters	Δρ_max_ = 0.79 e Å^−^^3^
0 restraints	Δρ_min_ = −0.78 e Å^−^^3^

### Special details

**Table:**

Geometry. All esds (except the esd in the dihedral angle between two l.s. planes) are estimated using the full covariance matrix. The cell esds are taken into account individually in the estimation of esds in distances, angles and torsion angles; correlations between esds in cell parameters are only used when they are defined by crystal symmetry. An approximate (isotropic) treatment of cell esds is used for estimating esds involving l.s. planes.
Refinement. Refinement of F^2^ against ALL reflections. The weighted R-factor wR and goodness of fit S are based on F^2^, conventional R-factors R are based on F, with F set to zero for negative F^2^. The threshold expression of F^2^ > 2sigma(F^2^) is used only for calculating R-factors(gt) etc. and is not relevant to the choice of reflections for refinement. R-factors based on F^2^ are statistically about twice as large as those based on F, and R- factors based on ALL data will be even larger.

### Fractional atomic coordinates and isotropic or equivalent isotropic displacement parameters (Å^2^)

**Table:**

	*x*	*y*	*z*	*U*_iso_*/*U*_eq_	
Mn2	0.62315 (4)	0.01645 (4)	0.122255 (15)	0.03700 (14)	
Mn1	−0.12571 (4)	0.21901 (4)	−0.119724 (14)	0.03640 (14)	
N1	−0.1268 (2)	0.0521 (2)	−0.12307 (8)	0.0420 (7)	
C1	−0.1725 (3)	0.0065 (3)	−0.15087 (11)	0.0464 (9)	
H1	−0.2060	0.0428	−0.1682	0.056*	
C2	−0.1722 (3)	−0.0915 (3)	−0.15496 (11)	0.0514 (10)	
H2	−0.2047	−0.1195	−0.1748	0.062*	
C3	−0.1239 (3)	−0.1482 (3)	−0.12975 (11)	0.0445 (9)	
C4	−0.0762 (3)	−0.1013 (3)	−0.10109 (11)	0.0473 (9)	
H4	−0.0421	−0.1360	−0.0835	0.057*	
C5	−0.0797 (3)	−0.0031 (3)	−0.09895 (11)	0.0472 (9)	
H5	−0.0474	0.0266	−0.0795	0.057*	
C6	−0.1261 (3)	−0.2528 (3)	−0.13367 (12)	0.0525 (10)	
H6	−0.1363	−0.2774	−0.1574	0.063*	
C7	−0.1148 (3)	−0.3143 (3)	−0.10635 (12)	0.0525 (10)	
H7	−0.1037	−0.2894	−0.0828	0.063*	
C8	−0.1182 (3)	−0.4190 (3)	−0.10983 (11)	0.0467 (9)	
C9	−0.1231 (3)	−0.4765 (3)	−0.07876 (11)	0.0540 (11)	
H9	−0.1237	−0.4492	−0.0552	0.065*	
C10	−0.1271 (3)	−0.5740 (3)	−0.08270 (11)	0.0527 (10)	
H10	−0.1311	−0.6113	−0.0614	0.063*	
C11	−0.1207 (3)	−0.5623 (3)	−0.14530 (12)	0.0549 (11)	
H11	−0.1197	−0.5915	−0.1685	0.066*	
C12	−0.1169 (3)	−0.4645 (3)	−0.14405 (11)	0.0537 (11)	
H12	−0.1135	−0.4291	−0.1658	0.064*	
N2	−0.1256 (2)	−0.6180 (2)	−0.11562 (9)	0.0483 (8)	
N21	0.0030 (2)	0.2156 (2)	−0.07933 (8)	0.0422 (7)	
C21	0.0906 (3)	0.2202 (4)	−0.09136 (12)	0.0589 (11)	
H21	0.0994	0.2310	−0.1165	0.071*	
C22	0.1680 (3)	0.2100 (4)	−0.06881 (13)	0.0675 (13)	
H22	0.2271	0.2160	−0.0788	0.081*	
C23	0.1600 (3)	0.1911 (3)	−0.03208 (13)	0.0558 (11)	
C24	0.0698 (3)	0.1901 (4)	−0.01895 (12)	0.0641 (13)	
H24	0.0600	0.1812	0.0062	0.077*	
C25	−0.0056 (3)	0.2024 (3)	−0.04298 (11)	0.0549 (11)	
H25	−0.0653	0.2014	−0.0333	0.066*	
C26	0.2465 (4)	0.1687 (4)	−0.01021 (14)	0.0691 (13)	
H26	0.3028	0.1894	−0.0198	0.083*	
C27	0.2501 (4)	0.1240 (4)	0.02013 (13)	0.0701 (13)	
H27	0.1935	0.1084	0.0308	0.084*	
C28	0.3371 (3)	0.0938 (3)	0.04045 (13)	0.0563 (11)	
C29	0.3289 (3)	0.0599 (5)	0.07585 (14)	0.0851 (18)	
H29	0.2701	0.0552	0.0862	0.102*	
C30	0.4062 (3)	0.0326 (4)	0.09612 (13)	0.0737 (15)	
H30	0.3978	0.0108	0.1202	0.088*	
C31	0.5007 (3)	0.0635 (3)	0.04844 (11)	0.0541 (11)	
H31	0.5595	0.0626	0.0380	0.065*	
C32	0.4251 (3)	0.0941 (3)	0.02680 (12)	0.0625 (12)	
H32	0.4347	0.1151	0.0027	0.075*	
N22	0.4927 (2)	0.0356 (2)	0.08342 (8)	0.0438 (7)	
N41	0.6187 (2)	−0.1488 (2)	0.11906 (9)	0.0433 (7)	
C41	0.5692 (3)	−0.1929 (3)	0.09229 (11)	0.0472 (9)	
H41	0.5347	−0.1556	0.0756	0.057*	
C42	0.5664 (3)	−0.2906 (3)	0.08807 (11)	0.0519 (10)	
H42	0.5309	−0.3174	0.0688	0.062*	
C43	0.6161 (3)	−0.3492 (3)	0.11222 (11)	0.0465 (9)	
C44	0.6675 (3)	−0.3037 (3)	0.14042 (12)	0.0519 (10)	
H44	0.7022	−0.3396	0.1575	0.062*	
C45	0.6666 (3)	−0.2057 (3)	0.14279 (12)	0.0497 (10)	
H45	0.7010	−0.1770	0.1619	0.060*	
C46	0.6144 (3)	−0.4537 (3)	0.10791 (12)	0.0527 (10)	
H46	0.6020	−0.4780	0.0843	0.063*	
C47	0.6291 (3)	−0.5155 (3)	0.13501 (12)	0.0527 (10)	
H47	0.6416	−0.4907	0.1585	0.063*	
C48	0.6277 (3)	−0.6200 (3)	0.13131 (11)	0.0464 (9)	
C49	0.6207 (3)	−0.6768 (3)	0.16241 (12)	0.0559 (11)	
H49	0.6161	−0.6492	0.1858	0.067*	
C50	0.6205 (3)	−0.7749 (3)	0.15851 (11)	0.0530 (11)	
H50	0.6145	−0.8118	0.1798	0.064*	
C51	0.6363 (3)	−0.7641 (3)	0.09618 (11)	0.0526 (10)	
H51	0.6431	−0.7935	0.0733	0.063*	
C52	0.6351 (3)	−0.6657 (3)	0.09734 (11)	0.0514 (10)	
H52	0.6393	−0.6303	0.0756	0.062*	
N42	0.6283 (2)	−0.8201 (2)	0.12618 (9)	0.0463 (8)	
N61	0.8742 (4)	0.4435 (5)	0.01294 (14)	0.1056 (18)	
C61	0.8781 (6)	0.5184 (5)	0.0353 (2)	0.111 (2)	
H61	0.8820	0.5785	0.0245	0.133*	
C62	0.8768 (5)	0.5137 (5)	0.07329 (16)	0.0887 (17)	
H62	0.8790	0.5696	0.0872	0.106*	
C63	0.8722 (3)	0.4262 (4)	0.09082 (13)	0.0657 (13)	
C64	0.8668 (4)	0.3469 (4)	0.06801 (14)	0.0739 (14)	
H64	0.8628	0.2858	0.0780	0.089*	
C65	0.8673 (5)	0.3602 (5)	0.02991 (16)	0.0937 (19)	
H65	0.8625	0.3060	0.0151	0.112*	
C66	0.8729 (3)	0.4229 (4)	0.13149 (14)	0.0674 (13)	
H66	0.8765	0.4813	0.1438	0.081*	
C67	0.8690 (4)	0.3472 (4)	0.15213 (14)	0.0709 (13)	
H67	0.8654	0.2886	0.1400	0.085*	
C68	0.8698 (4)	0.3450 (4)	0.19293 (13)	0.0708 (14)	
C69	0.8767 (5)	0.4244 (5)	0.21498 (16)	0.100 (2)	
H69	0.8812	0.4847	0.2043	0.120*	
C70	0.8771 (6)	0.4151 (6)	0.25317 (18)	0.116 (3)	
H70	0.8818	0.4705	0.2673	0.139*	
C71	0.8637 (6)	0.2587 (6)	0.24944 (19)	0.119 (3)	
H71	0.8588	0.1994	0.2609	0.143*	
C72	0.8622 (6)	0.2603 (5)	0.21140 (16)	0.103 (2)	
H72	0.8559	0.2036	0.1982	0.124*	
N62	0.8713 (4)	0.3331 (5)	0.27070 (14)	0.1031 (17)	
N81	−0.2528 (2)	0.2278 (2)	−0.16142 (9)	0.0448 (8)	
C81	−0.3403 (3)	0.2265 (4)	−0.14929 (13)	0.0637 (13)	
H81	−0.3490	0.2250	−0.1238	0.076*	
C82	−0.4178 (3)	0.2272 (4)	−0.17205 (14)	0.0758 (15)	
H82	−0.4768	0.2269	−0.1617	0.091*	
C83	−0.4103 (3)	0.2284 (4)	−0.20947 (14)	0.0634 (12)	
C84	−0.3201 (4)	0.2293 (4)	−0.22283 (12)	0.0635 (12)	
H84	−0.3104	0.2298	−0.2483	0.076*	
C85	−0.2444 (3)	0.2295 (3)	−0.19821 (11)	0.0545 (11)	
H85	−0.1846	0.2309	−0.2078	0.065*	
C86	−0.4971 (4)	0.2283 (4)	−0.23282 (13)	0.0800 (16)	
H86	−0.5532	0.2283	−0.2203	0.096*	
N101	−0.3906 (4)	−0.4425 (5)	0.00920 (14)	0.0989 (16)	
C101	−0.3648 (5)	−0.5210 (6)	−0.00842 (18)	0.100 (2)	
H101	−0.3497	−0.5746	0.0057	0.120*	
C102	−0.3589 (4)	−0.5281 (5)	−0.04643 (17)	0.0925 (18)	
H102	−0.3421	−0.5856	−0.0573	0.111*	
C103	−0.3780 (4)	−0.4503 (5)	−0.06783 (15)	0.0788 (16)	
C104	−0.4056 (5)	−0.3687 (5)	−0.04989 (17)	0.0963 (19)	
H104	−0.4208	−0.3142	−0.0635	0.116*	
C105	−0.4109 (5)	−0.3675 (6)	−0.01181 (18)	0.103 (2)	
H105	−0.4295	−0.3113	−0.0003	0.123*	
C106	−0.3716 (4)	−0.4604 (6)	−0.10878 (17)	0.097 (2)	
H106	−0.3609	−0.5218	−0.1176	0.116*	
C107	−0.3786 (4)	−0.3983 (6)	−0.13192 (19)	0.105 (2)	
H107	−0.3873	−0.3365	−0.1232	0.126*	
C108	−0.3749 (4)	−0.4100 (6)	−0.17381 (17)	0.096 (2)	
C109	−0.3663 (6)	−0.4953 (6)	−0.1922 (2)	0.122 (3)	
H109	−0.3608	−0.5520	−0.1788	0.146*	
C110	−0.3657 (7)	−0.4969 (6)	−0.2306 (2)	0.135 (3)	
H110	−0.3599	−0.5558	−0.2422	0.161*	
C111	−0.3801 (5)	−0.3401 (6)	−0.23341 (19)	0.106 (2)	
H111	−0.3854	−0.2844	−0.2474	0.127*	
N102	−0.3728 (5)	−0.4211 (5)	−0.25138 (15)	0.115 (2)	
N121	0.7525 (2)	0.0070 (2)	0.16209 (8)	0.0428 (7)	
C121	0.7446 (3)	−0.0039 (3)	0.19864 (11)	0.0541 (11)	
H121	0.6849	−0.0061	0.2084	0.065*	
C122	0.8207 (3)	−0.0121 (3)	0.22282 (12)	0.0602 (12)	
H122	0.8114	−0.0200	0.2481	0.072*	
C123	0.9097 (3)	−0.0085 (3)	0.20951 (12)	0.0548 (11)	
C124	0.9175 (3)	0.0022 (4)	0.17212 (13)	0.0725 (15)	
H124	0.9764	0.0049	0.1617	0.087*	
C125	0.8391 (3)	0.0092 (4)	0.14988 (12)	0.0644 (13)	
H125	0.8472	0.0159	0.1245	0.077*	
C126	0.9966 (4)	−0.0124 (4)	0.23262 (12)	0.0684 (13)	
H126	1.0524	−0.0152	0.2199	0.082*	
N13	−0.0334 (3)	0.2265 (3)	−0.16706 (9)	0.0530 (9)	
C13	0.0280 (3)	0.2525 (3)	−0.18490 (11)	0.0493 (10)	
Se13	0.12347 (4)	0.29280 (5)	−0.211172 (17)	0.0860 (2)	
N14	−0.2189 (2)	0.2092 (3)	−0.07297 (9)	0.0523 (8)	
C14	−0.2774 (3)	0.2041 (3)	−0.05138 (11)	0.0475 (9)	
Se14	−0.36807 (4)	0.19698 (5)	−0.018923 (14)	0.07474 (17)	
N16	0.5330 (3)	0.0107 (3)	0.16991 (9)	0.0547 (9)	
C16	0.4719 (3)	0.0262 (3)	0.18959 (11)	0.0503 (10)	
Se16	0.37749 (4)	0.05023 (6)	0.219058 (15)	0.0860 (2)	
N17	0.7137 (2)	0.0225 (3)	0.07427 (9)	0.0511 (8)	
C17	0.7777 (3)	0.0433 (3)	0.05675 (11)	0.0459 (9)	
Se17	0.87743 (3)	0.07271 (4)	0.030650 (14)	0.06694 (15)	
C112	−0.3805 (5)	−0.3312 (6)	−0.19489 (18)	0.106 (2)	
H112	−0.3846	−0.2712	−0.1839	0.128*	

### Atomic displacement parameters (Å^2^)

**Table:**

	*U*^11^	*U*^22^	*U*^33^	*U*^12^	*U*^13^	*U*^23^
Mn2	0.0391 (3)	0.0353 (3)	0.0365 (3)	0.0009 (2)	−0.0034 (2)	0.0015 (2)
Mn1	0.0385 (3)	0.0337 (3)	0.0368 (3)	0.0003 (2)	−0.0042 (2)	0.0031 (2)
N1	0.0491 (18)	0.0320 (16)	0.0449 (17)	0.0005 (14)	0.0006 (14)	0.0011 (14)
C1	0.058 (3)	0.033 (2)	0.048 (2)	0.0017 (17)	−0.0094 (19)	0.0031 (17)
C2	0.061 (3)	0.044 (2)	0.049 (2)	0.000 (2)	−0.0102 (19)	−0.0026 (18)
C3	0.048 (2)	0.034 (2)	0.051 (2)	−0.0010 (17)	0.0011 (18)	0.0024 (17)
C4	0.053 (2)	0.037 (2)	0.052 (2)	0.0006 (18)	−0.0081 (19)	0.0053 (18)
C5	0.057 (2)	0.036 (2)	0.048 (2)	−0.0041 (18)	−0.0057 (19)	0.0028 (17)
C6	0.065 (3)	0.039 (2)	0.054 (2)	0.000 (2)	−0.009 (2)	−0.0029 (19)
C7	0.068 (3)	0.039 (2)	0.051 (2)	−0.002 (2)	−0.003 (2)	−0.0034 (19)
C8	0.057 (2)	0.037 (2)	0.046 (2)	−0.0006 (18)	−0.0037 (18)	0.0013 (17)
C9	0.077 (3)	0.044 (2)	0.041 (2)	−0.001 (2)	−0.004 (2)	−0.0001 (18)
C10	0.077 (3)	0.039 (2)	0.042 (2)	−0.001 (2)	−0.002 (2)	0.0059 (18)
C11	0.081 (3)	0.038 (2)	0.046 (2)	0.000 (2)	−0.007 (2)	0.0013 (18)
C12	0.079 (3)	0.035 (2)	0.047 (2)	−0.001 (2)	0.000 (2)	0.0063 (18)
N2	0.060 (2)	0.0342 (17)	0.0508 (19)	−0.0013 (15)	−0.0068 (16)	0.0038 (15)
N21	0.0422 (18)	0.0410 (18)	0.0431 (17)	0.0009 (14)	−0.0068 (14)	0.0045 (14)
C21	0.048 (3)	0.077 (3)	0.052 (2)	0.000 (2)	−0.004 (2)	0.005 (2)
C22	0.045 (3)	0.092 (4)	0.065 (3)	0.005 (2)	−0.008 (2)	0.004 (3)
C23	0.049 (2)	0.048 (3)	0.070 (3)	0.0023 (19)	−0.017 (2)	0.006 (2)
C24	0.071 (3)	0.077 (3)	0.044 (2)	−0.007 (3)	−0.017 (2)	0.014 (2)
C25	0.049 (2)	0.071 (3)	0.044 (2)	−0.001 (2)	−0.0067 (18)	0.007 (2)
C26	0.064 (3)	0.076 (3)	0.067 (3)	−0.011 (3)	−0.012 (2)	0.009 (3)
C27	0.063 (3)	0.086 (4)	0.061 (3)	0.002 (3)	−0.012 (2)	0.006 (3)
C28	0.049 (2)	0.057 (3)	0.062 (3)	0.003 (2)	−0.017 (2)	0.003 (2)
C29	0.045 (3)	0.151 (6)	0.060 (3)	0.017 (3)	−0.003 (2)	0.013 (3)
C30	0.047 (3)	0.124 (5)	0.050 (2)	0.010 (3)	−0.003 (2)	0.015 (3)
C31	0.047 (2)	0.070 (3)	0.046 (2)	−0.004 (2)	−0.0049 (18)	0.012 (2)
C32	0.072 (3)	0.069 (3)	0.045 (2)	−0.009 (2)	−0.017 (2)	0.017 (2)
N22	0.0393 (18)	0.050 (2)	0.0424 (17)	0.0013 (14)	−0.0058 (14)	0.0040 (15)
N41	0.0440 (18)	0.0382 (17)	0.0475 (18)	0.0006 (14)	−0.0004 (14)	0.0017 (14)
C41	0.057 (2)	0.038 (2)	0.047 (2)	0.0035 (18)	−0.0054 (18)	0.0042 (17)
C42	0.060 (3)	0.046 (2)	0.048 (2)	−0.002 (2)	−0.0099 (19)	−0.0002 (19)
C43	0.052 (2)	0.040 (2)	0.048 (2)	0.0006 (18)	0.0020 (18)	−0.0028 (18)
C44	0.058 (3)	0.037 (2)	0.060 (2)	−0.0020 (19)	−0.012 (2)	0.0058 (19)
C45	0.055 (2)	0.040 (2)	0.054 (2)	−0.0036 (19)	−0.0102 (19)	0.0029 (19)
C46	0.063 (3)	0.039 (2)	0.055 (2)	0.003 (2)	−0.008 (2)	−0.0049 (19)
C47	0.061 (3)	0.043 (2)	0.055 (2)	−0.002 (2)	0.000 (2)	−0.004 (2)
C48	0.054 (2)	0.032 (2)	0.053 (2)	0.0029 (17)	−0.0031 (19)	0.0003 (17)
C49	0.079 (3)	0.042 (2)	0.046 (2)	0.002 (2)	0.001 (2)	−0.0072 (19)
C50	0.077 (3)	0.042 (2)	0.040 (2)	−0.001 (2)	0.001 (2)	0.0044 (18)
C51	0.075 (3)	0.042 (2)	0.041 (2)	0.002 (2)	−0.004 (2)	−0.0037 (18)
C52	0.073 (3)	0.037 (2)	0.044 (2)	−0.002 (2)	0.000 (2)	0.0043 (17)
N42	0.059 (2)	0.0347 (17)	0.0454 (18)	−0.0002 (15)	−0.0045 (15)	−0.0010 (14)
N61	0.135 (5)	0.115 (5)	0.066 (3)	0.001 (4)	0.005 (3)	0.020 (3)
C61	0.159 (7)	0.083 (5)	0.090 (5)	−0.016 (5)	−0.005 (5)	0.021 (4)
C62	0.115 (5)	0.080 (4)	0.071 (4)	−0.012 (4)	−0.003 (3)	−0.001 (3)
C63	0.059 (3)	0.079 (4)	0.059 (3)	−0.001 (2)	0.003 (2)	0.004 (3)
C64	0.087 (4)	0.071 (3)	0.064 (3)	0.000 (3)	0.010 (3)	0.000 (3)
C65	0.111 (5)	0.100 (5)	0.071 (4)	0.009 (4)	0.009 (3)	−0.009 (3)
C66	0.066 (3)	0.073 (3)	0.064 (3)	−0.003 (3)	0.000 (2)	−0.005 (3)
C67	0.076 (3)	0.074 (4)	0.063 (3)	−0.002 (3)	−0.002 (2)	−0.005 (3)
C68	0.072 (3)	0.086 (4)	0.054 (3)	0.003 (3)	−0.002 (2)	−0.005 (3)
C69	0.149 (6)	0.085 (4)	0.065 (3)	0.003 (4)	−0.005 (4)	−0.002 (3)
C70	0.173 (8)	0.103 (6)	0.071 (4)	0.007 (5)	−0.004 (4)	−0.019 (4)
C71	0.191 (9)	0.095 (5)	0.072 (4)	0.010 (5)	0.003 (5)	0.015 (4)
C72	0.175 (7)	0.069 (4)	0.064 (3)	0.000 (4)	−0.007 (4)	0.001 (3)
N62	0.135 (5)	0.111 (5)	0.063 (3)	0.009 (4)	−0.002 (3)	0.002 (3)
N81	0.0441 (19)	0.046 (2)	0.0434 (17)	−0.0011 (15)	−0.0091 (14)	0.0050 (14)
C81	0.047 (3)	0.092 (4)	0.052 (2)	−0.002 (2)	−0.006 (2)	0.012 (2)
C82	0.045 (3)	0.117 (5)	0.065 (3)	−0.005 (3)	−0.011 (2)	0.016 (3)
C83	0.048 (3)	0.073 (3)	0.068 (3)	−0.005 (2)	−0.016 (2)	0.008 (2)
C84	0.072 (3)	0.074 (3)	0.044 (2)	−0.002 (3)	−0.015 (2)	0.005 (2)
C85	0.053 (3)	0.064 (3)	0.047 (2)	0.001 (2)	−0.0084 (19)	0.005 (2)
C86	0.066 (3)	0.110 (5)	0.063 (3)	−0.009 (3)	−0.013 (3)	0.012 (3)
N101	0.109 (4)	0.121 (5)	0.067 (3)	−0.005 (4)	0.000 (3)	−0.011 (3)
C101	0.101 (5)	0.119 (6)	0.081 (4)	0.013 (4)	0.009 (4)	0.025 (4)
C102	0.092 (4)	0.104 (5)	0.083 (4)	0.012 (4)	0.011 (3)	0.000 (4)
C103	0.058 (3)	0.115 (5)	0.063 (3)	−0.009 (3)	0.005 (2)	−0.006 (3)
C104	0.116 (5)	0.092 (5)	0.081 (4)	−0.002 (4)	0.000 (4)	0.011 (4)
C105	0.130 (6)	0.099 (5)	0.078 (4)	−0.002 (4)	0.002 (4)	−0.014 (4)
C106	0.083 (4)	0.131 (6)	0.076 (4)	−0.007 (4)	0.003 (3)	0.021 (4)
C107	0.074 (4)	0.148 (7)	0.093 (5)	−0.020 (4)	−0.004 (3)	0.025 (5)
C108	0.078 (4)	0.143 (7)	0.067 (4)	−0.019 (4)	−0.004 (3)	0.015 (4)
C109	0.164 (8)	0.118 (6)	0.082 (5)	−0.013 (5)	−0.018 (5)	0.050 (5)
C110	0.209 (10)	0.105 (6)	0.089 (5)	−0.010 (6)	−0.014 (6)	0.022 (4)
C111	0.119 (6)	0.111 (6)	0.086 (5)	−0.003 (5)	0.002 (4)	0.036 (4)
N102	0.152 (6)	0.119 (5)	0.073 (3)	0.003 (4)	−0.003 (3)	0.017 (4)
N121	0.0401 (18)	0.0459 (19)	0.0419 (17)	0.0007 (14)	−0.0073 (14)	−0.0015 (14)
C121	0.045 (2)	0.071 (3)	0.046 (2)	−0.001 (2)	−0.0069 (18)	0.003 (2)
C122	0.072 (3)	0.068 (3)	0.040 (2)	−0.002 (2)	−0.014 (2)	0.002 (2)
C123	0.045 (2)	0.061 (3)	0.058 (3)	0.004 (2)	−0.010 (2)	−0.003 (2)
C124	0.045 (3)	0.117 (5)	0.055 (3)	0.004 (3)	−0.006 (2)	−0.003 (3)
C125	0.047 (3)	0.098 (4)	0.048 (2)	0.003 (2)	−0.007 (2)	−0.001 (2)
C126	0.059 (3)	0.093 (4)	0.053 (2)	0.006 (3)	−0.006 (2)	−0.005 (2)
N13	0.051 (2)	0.063 (2)	0.0445 (18)	0.0014 (18)	0.0033 (16)	0.0029 (17)
C13	0.055 (3)	0.050 (2)	0.043 (2)	0.006 (2)	−0.0079 (19)	−0.0003 (18)
Se13	0.0671 (3)	0.1123 (5)	0.0793 (4)	−0.0190 (3)	0.0135 (3)	0.0173 (3)
N14	0.052 (2)	0.059 (2)	0.0462 (19)	−0.0037 (17)	0.0025 (16)	0.0048 (16)
C14	0.054 (2)	0.047 (2)	0.041 (2)	−0.0002 (19)	−0.0088 (19)	0.0046 (18)
Se14	0.0579 (3)	0.1092 (5)	0.0576 (3)	0.0026 (3)	0.0133 (2)	0.0142 (3)
N16	0.053 (2)	0.067 (2)	0.0444 (19)	−0.0019 (18)	0.0019 (17)	0.0009 (17)
C16	0.053 (3)	0.054 (3)	0.043 (2)	−0.002 (2)	−0.010 (2)	0.0060 (19)
Se16	0.0584 (3)	0.1393 (6)	0.0605 (3)	0.0185 (3)	0.0091 (2)	−0.0018 (3)
N17	0.052 (2)	0.058 (2)	0.0436 (18)	0.0064 (17)	0.0037 (16)	0.0018 (16)
C17	0.055 (2)	0.041 (2)	0.041 (2)	0.0048 (19)	−0.0081 (19)	0.0016 (17)
Se17	0.0544 (3)	0.0778 (4)	0.0687 (3)	−0.0104 (2)	0.0053 (2)	0.0131 (3)
C112	0.113 (5)	0.129 (6)	0.078 (4)	−0.003 (5)	0.007 (4)	0.018 (4)

### Geometric parameters (Å, °)

**Table:**

Mn2—N16	2.172 (4)	C52—H52	0.9300
Mn2—N17	2.185 (3)	N42—Mn2^ii^	2.293 (3)
Mn2—N42^i^	2.293 (3)	N61—C65	1.320 (8)
Mn2—N41	2.317 (3)	N61—C61	1.323 (9)
Mn2—N121	2.322 (3)	C61—C62	1.371 (9)
Mn2—N22	2.325 (3)	C61—H61	0.9300
Mn1—N14	2.176 (3)	C62—C63	1.380 (8)
Mn1—N13	2.183 (4)	C62—H62	0.9300
Mn1—N2^i^	2.285 (3)	C63—C64	1.382 (7)
Mn1—N21	2.324 (3)	C63—C66	1.467 (7)
Mn1—N1	2.338 (3)	C64—C65	1.387 (7)
Mn1—N81	2.339 (3)	C64—H64	0.9300
N1—C5	1.335 (5)	C65—H65	0.9300
N1—C1	1.346 (5)	C66—C67	1.297 (7)
C1—C2	1.380 (6)	C66—H66	0.9300
C1—H1	0.9300	C67—C68	1.471 (7)
C2—C3	1.381 (6)	C67—H67	0.9300
C2—H2	0.9300	C68—C72	1.364 (8)
C3—C4	1.392 (5)	C68—C69	1.369 (8)
C3—C6	1.470 (6)	C69—C70	1.383 (8)
C4—C5	1.377 (5)	C69—H69	0.9300
C4—H4	0.9300	C70—N62	1.314 (9)
C5—H5	0.9300	C70—H70	0.9300
C6—C7	1.315 (6)	C71—N62	1.296 (9)
C6—H6	0.9300	C71—C72	1.371 (8)
C7—C8	1.472 (6)	C71—H71	0.9300
C7—H7	0.9300	C72—H72	0.9300
C8—C9	1.383 (6)	N81—C81	1.335 (5)
C8—C12	1.389 (6)	N81—C85	1.335 (5)
C9—C10	1.373 (6)	C81—C82	1.366 (6)
C9—H9	0.9300	C81—H81	0.9300
C10—N2	1.338 (5)	C82—C83	1.356 (7)
C10—H10	0.9300	C82—H82	0.9300
C11—N2	1.328 (5)	C83—C84	1.387 (7)
C11—C12	1.369 (6)	C83—C86	1.486 (7)
C11—H11	0.9300	C84—C85	1.386 (6)
C12—H12	0.9300	C84—H84	0.9300
N2—Mn1^ii^	2.285 (3)	C85—H85	0.9300
N21—C25	1.332 (5)	C86—C86^iii^	1.240 (10)
N21—C21	1.336 (5)	C86—H86	0.9300
C21—C22	1.368 (6)	N101—C105	1.323 (8)
C21—H21	0.9300	N101—C101	1.326 (9)
C22—C23	1.358 (6)	C101—C102	1.379 (8)
C22—H22	0.9300	C101—H101	0.9300
C23—C24	1.384 (7)	C102—C103	1.359 (9)
C23—C26	1.486 (6)	C102—H102	0.9300
C24—C25	1.380 (6)	C103—C104	1.375 (9)
C24—H24	0.9300	C103—C106	1.488 (8)
C25—H25	0.9300	C104—C105	1.377 (8)
C26—C27	1.260 (7)	C104—H104	0.9300
C26—H26	0.9300	C105—H105	0.9300
C27—C28	1.492 (6)	C106—C107	1.208 (9)
C27—H27	0.9300	C106—H106	0.9300
C28—C32	1.362 (6)	C107—C108	1.522 (9)
C28—C29	1.370 (7)	C107—H107	0.9300
C29—C30	1.367 (6)	C108—C112	1.341 (9)
C29—H29	0.9300	C108—C109	1.372 (10)
C30—N22	1.330 (5)	C109—C110	1.385 (10)
C30—H30	0.9300	C109—H109	0.9300
C31—N22	1.328 (5)	C110—N102	1.302 (9)
C31—C32	1.388 (6)	C110—H110	0.9300
C31—H31	0.9300	C111—N102	1.310 (9)
C32—H32	0.9300	C111—C112	1.395 (9)
N41—C41	1.337 (5)	C111—H111	0.9300
N41—C45	1.345 (5)	N121—C125	1.325 (5)
C41—C42	1.376 (6)	N121—C121	1.334 (5)
C41—H41	0.9300	C121—C122	1.386 (6)
C42—C43	1.381 (6)	C121—H121	0.9300
C42—H42	0.9300	C122—C123	1.372 (6)
C43—C44	1.396 (6)	C122—H122	0.9300
C43—C46	1.471 (6)	C123—C124	1.363 (6)
C44—C45	1.375 (6)	C123—C126	1.483 (6)
C44—H44	0.9300	C124—C125	1.369 (6)
C45—H45	0.9300	C124—H124	0.9300
C46—C47	1.318 (6)	C125—H125	0.9300
C46—H46	0.9300	C126—C126^iv^	1.256 (9)
C47—C48	1.469 (6)	C126—H126	0.9300
C47—H47	0.9300	N13—C13	1.158 (5)
C48—C49	1.379 (6)	C13—Se13	1.771 (5)
C48—C52	1.388 (5)	N14—C14	1.157 (5)
C49—C50	1.381 (6)	C14—Se14	1.769 (4)
C49—H49	0.9300	N16—C16	1.158 (5)
C50—N42	1.333 (5)	C16—Se16	1.769 (5)
C50—H50	0.9300	N17—C17	1.161 (5)
C51—N42	1.343 (5)	C17—Se17	1.774 (4)
C51—C52	1.378 (6)	C112—H112	0.9300
C51—H51	0.9300		
			
N16—Mn2—N17	179.89 (16)	C48—C49—C50	119.3 (4)
N16—Mn2—N42^i^	90.39 (13)	C48—C49—H49	120.3
N17—Mn2—N42^i^	89.53 (13)	C50—C49—H49	120.3
N16—Mn2—N41	89.22 (13)	N42—C50—C49	124.1 (4)
N17—Mn2—N41	90.87 (12)	N42—C50—H50	117.9
N42^i^—Mn2—N41	179.27 (12)	C49—C50—H50	117.9
N16—Mn2—N121	89.27 (12)	N42—C51—C52	123.9 (4)
N17—Mn2—N121	90.81 (12)	N42—C51—H51	118.1
N42^i^—Mn2—N121	89.70 (12)	C52—C51—H51	118.1
N41—Mn2—N121	89.68 (11)	C51—C52—C48	119.2 (4)
N16—Mn2—N22	89.90 (12)	C51—C52—H52	120.4
N17—Mn2—N22	90.01 (12)	C48—C52—H52	120.4
N42^i^—Mn2—N22	86.94 (12)	C50—N42—C51	116.0 (3)
N41—Mn2—N22	93.68 (12)	C50—N42—Mn2^ii^	121.6 (3)
N121—Mn2—N22	176.53 (12)	C51—N42—Mn2^ii^	122.4 (3)
N14—Mn1—N13	178.94 (14)	C65—N61—C61	114.7 (6)
N14—Mn1—N2^i^	90.72 (13)	N61—C61—C62	124.8 (6)
N13—Mn1—N2^i^	90.19 (13)	N61—C61—H61	117.6
N14—Mn1—N21	90.20 (12)	C62—C61—H61	117.6
N13—Mn1—N21	90.37 (12)	C61—C62—C63	120.1 (6)
N2^i^—Mn1—N21	88.85 (12)	C61—C62—H62	119.9
N14—Mn1—N1	88.46 (12)	C63—C62—H62	119.9
N13—Mn1—N1	90.63 (13)	C62—C63—C64	116.2 (5)
N2^i^—Mn1—N1	179.16 (12)	C62—C63—C66	119.1 (5)
N21—Mn1—N1	90.96 (11)	C64—C63—C66	124.6 (5)
N14—Mn1—N81	91.17 (12)	C63—C64—C65	118.7 (6)
N13—Mn1—N81	88.29 (12)	C63—C64—H64	120.7
N2^i^—Mn1—N81	89.37 (12)	C65—C64—H64	120.7
N21—Mn1—N81	177.77 (11)	N61—C65—C64	125.4 (6)
N1—Mn1—N81	90.84 (11)	N61—C65—H65	117.3
C5—N1—C1	116.2 (3)	C64—C65—H65	117.3
C5—N1—Mn1	122.8 (3)	C67—C66—C63	126.9 (5)
C1—N1—Mn1	121.0 (2)	C67—C66—H66	116.6
N1—C1—C2	123.3 (4)	C63—C66—H66	116.6
N1—C1—H1	118.4	C66—C67—C68	126.3 (5)
C2—C1—H1	118.4	C66—C67—H67	116.9
C1—C2—C3	120.2 (4)	C68—C67—H67	116.9
C1—C2—H2	119.9	C72—C68—C69	115.3 (5)
C3—C2—H2	119.9	C72—C68—C67	120.5 (5)
C2—C3—C4	116.7 (4)	C69—C68—C67	124.3 (5)
C2—C3—C6	119.9 (4)	C68—C69—C70	120.1 (6)
C4—C3—C6	123.4 (4)	C68—C69—H69	120.0
C5—C4—C3	119.6 (4)	C70—C69—H69	120.0
C5—C4—H4	120.2	N62—C70—C69	124.2 (7)
C3—C4—H4	120.2	N62—C70—H70	117.9
N1—C5—C4	124.0 (4)	C69—C70—H70	117.9
N1—C5—H5	118.0	N62—C71—C72	125.3 (7)
C4—C5—H5	118.0	N62—C71—H71	117.4
C7—C6—C3	125.2 (4)	C72—C71—H71	117.4
C7—C6—H6	117.4	C68—C72—C71	120.2 (6)
C3—C6—H6	117.4	C68—C72—H72	119.9
C6—C7—C8	125.8 (4)	C71—C72—H72	119.9
C6—C7—H7	117.1	C71—N62—C70	115.0 (6)
C8—C7—H7	117.1	C81—N81—C85	115.6 (4)
C9—C8—C12	117.1 (4)	C81—N81—Mn1	120.7 (3)
C9—C8—C7	120.8 (4)	C85—N81—Mn1	123.6 (3)
C12—C8—C7	122.1 (4)	N81—C81—C82	123.9 (4)
C10—C9—C8	119.8 (4)	N81—C81—H81	118.0
C10—C9—H9	120.1	C82—C81—H81	118.0
C8—C9—H9	120.1	C83—C82—C81	121.2 (5)
N2—C10—C9	123.2 (4)	C83—C82—H82	119.4
N2—C10—H10	118.4	C81—C82—H82	119.4
C9—C10—H10	118.4	C82—C83—C84	116.1 (4)
N2—C11—C12	124.3 (4)	C82—C83—C86	118.8 (5)
N2—C11—H11	117.9	C84—C83—C86	125.2 (5)
C12—C11—H11	117.9	C85—C84—C83	119.9 (4)
C11—C12—C8	119.1 (4)	C85—C84—H84	120.1
C11—C12—H12	120.5	C83—C84—H84	120.1
C8—C12—H12	120.5	N81—C85—C84	123.4 (4)
C11—N2—C10	116.5 (3)	N81—C85—H85	118.3
C11—N2—Mn1^ii^	122.3 (3)	C84—C85—H85	118.3
C10—N2—Mn1^ii^	121.1 (3)	C86^iii^—C86—C83	127.2 (7)
C25—N21—C21	115.7 (3)	C86^iii^—C86—H86	116.4
C25—N21—Mn1	122.1 (3)	C83—C86—H86	116.4
C21—N21—Mn1	122.1 (3)	C105—N101—C101	116.3 (6)
N21—C21—C22	123.7 (4)	N101—C101—C102	124.1 (7)
N21—C21—H21	118.1	N101—C101—H101	118.0
C22—C21—H21	118.1	C102—C101—H101	118.0
C23—C22—C21	121.1 (5)	C103—C102—C101	119.4 (7)
C23—C22—H22	119.5	C103—C102—H102	120.3
C21—C22—H22	119.5	C101—C102—H102	120.3
C22—C23—C24	115.7 (4)	C102—C103—C104	117.1 (5)
C22—C23—C26	118.2 (4)	C102—C103—C106	118.1 (6)
C24—C23—C26	126.0 (4)	C104—C103—C106	124.8 (6)
C25—C24—C23	120.4 (4)	C103—C104—C105	120.1 (6)
C25—C24—H24	119.8	C103—C104—H104	120.0
C23—C24—H24	119.8	C105—C104—H104	120.0
N21—C25—C24	123.2 (4)	N101—C105—C104	123.1 (7)
N21—C25—H25	118.4	N101—C105—H105	118.5
C24—C25—H25	118.4	C104—C105—H105	118.5
C27—C26—C23	125.7 (5)	C107—C106—C103	127.7 (8)
C27—C26—H26	117.1	C107—C106—H106	116.2
C23—C26—H26	117.1	C103—C106—H106	116.2
C26—C27—C28	125.8 (5)	C106—C107—C108	127.2 (9)
C26—C27—H27	117.1	C106—C107—H107	116.4
C28—C27—H27	117.1	C108—C107—H107	116.4
C32—C28—C29	115.8 (4)	C112—C108—C109	116.6 (6)
C32—C28—C27	126.2 (4)	C112—C108—C107	118.1 (8)
C29—C28—C27	118.0 (4)	C109—C108—C107	125.3 (7)
C30—C29—C28	120.8 (5)	C108—C109—C110	119.9 (7)
C30—C29—H29	119.6	C108—C109—H109	120.0
C28—C29—H29	119.6	C110—C109—H109	120.0
N22—C30—C29	123.8 (4)	N102—C110—C109	124.1 (8)
N22—C30—H30	118.1	N102—C110—H110	117.9
C29—C30—H30	118.1	C109—C110—H110	117.9
N22—C31—C32	123.0 (4)	N102—C111—C112	124.9 (7)
N22—C31—H31	118.5	N102—C111—H111	117.6
C32—C31—H31	118.5	C112—C111—H111	117.6
C28—C32—C31	120.8 (4)	C110—N102—C111	115.2 (6)
C28—C32—H32	119.6	C125—N121—C121	115.5 (3)
C31—C32—H32	119.6	C125—N121—Mn2	122.2 (3)
C31—N22—C30	115.8 (4)	C121—N121—Mn2	122.3 (3)
C31—N22—Mn2	121.5 (3)	N121—C121—C122	123.3 (4)
C30—N22—Mn2	122.0 (3)	N121—C121—H121	118.3
C41—N41—C45	116.2 (3)	C122—C121—H121	118.3
C41—N41—Mn2	120.7 (3)	C123—C122—C121	120.0 (4)
C45—N41—Mn2	123.1 (3)	C123—C122—H122	120.0
N41—C41—C42	123.5 (4)	C121—C122—H122	120.0
N41—C41—H41	118.2	C124—C123—C122	116.5 (4)
C42—C41—H41	118.2	C124—C123—C126	118.3 (4)
C41—C42—C43	120.4 (4)	C122—C123—C126	125.1 (4)
C41—C42—H42	119.8	C123—C124—C125	120.3 (5)
C43—C42—H42	119.8	C123—C124—H124	119.9
C42—C43—C44	116.5 (4)	C125—C124—H124	119.9
C42—C43—C46	121.1 (4)	N121—C125—C124	124.4 (4)
C44—C43—C46	122.5 (4)	N121—C125—H125	117.8
C45—C44—C43	119.6 (4)	C124—C125—H125	117.8
C45—C44—H44	120.2	C126^iv^—C126—C123	127.4 (6)
C43—C44—H44	120.2	C126^iv^—C126—H126	116.3
N41—C45—C44	123.8 (4)	C123—C126—H126	116.3
N41—C45—H45	118.1	C13—N13—Mn1	158.1 (3)
C44—C45—H45	118.1	N13—C13—Se13	178.6 (4)
C47—C46—C43	124.8 (4)	C14—N14—Mn1	171.5 (3)
C47—C46—H46	117.6	N14—C14—Se14	179.1 (4)
C43—C46—H46	117.6	C16—N16—Mn2	161.2 (3)
C46—C47—C48	125.7 (4)	N16—C16—Se16	179.1 (4)
C46—C47—H47	117.1	C17—N17—Mn2	158.2 (3)
C48—C47—H47	117.1	N17—C17—Se17	178.4 (4)
C49—C48—C52	117.4 (4)	C108—C112—C111	119.3 (8)
C49—C48—C47	120.0 (4)	C108—C112—H112	120.4
C52—C48—C47	122.5 (4)	C111—C112—H112	120.4

Symmetry codes: (i) *x*, *y*+1, *z*; (ii) *x*, *y*−1, *z*; (iii) −*x*−1, *y*, −*z*−1/2; (iv) −*x*+2, *y*, −*z*+1/2.
